# Atlanta Medical College

**Published:** 1859-06

**Authors:** 


### ATLANTA MEDICAL COLLEGE.

This Institution has continued to grow in the confidence of the medical profession, and has now in attendance upon the regular course of Lectures, a larger number than at any former period.

Among the class are found the representatives of nine States, and, in addition to this, it may be stated, as the most gratifying feature connected with the matter, in appearance and general conduct, are in marked (favorable) contrast with the medical classes, that occasionally assemble in the oldest colleges of the country. The object of the present class is manifestly to use the opportunities afforded for the acquisition of medical knowledge, to the best advantage.

---

